# Chronic Rejection Pathology after Orthotopic Lung Transplantation in Mice: The Development of a Murine BOS Model and Its Drawbacks

**DOI:** 10.1371/journal.pone.0029802

**Published:** 2012-01-06

**Authors:** Stéphanie De Vleeschauwer, Wolfgang Jungraithmayr, Shana Wauters, Stijn Willems, Manuela Rinaldi, Annemie Vaneylen, Stijn Verleden, Anna Willems-Widyastuti, Ken Bracke, Guy Brusselle, Erik Verbeken, Dirk Van Raemdonck, Geert Verleden, Bart Vanaudenaerde

**Affiliations:** 1 Laboratory of Pneumology, Katholieke Universiteit Leuven, Leuven, Belgium; 2 Laboratory of Experimental Thoracic Surgery, Katholieke Universiteit Leuven, Leuven, Belgium; 3 Department of Pneumology, University Hospitals Leuven, Leuven, Belgium; 4 Department of Thoracic Surgery, University Hospitals Leuven, Leuven, Belgium; 5 Department of Pathology, University Hospitals Leuven, Leuven, Belgium; 6 Laboratory of Thoracic Surgery, University Hospital, Zurich, Switzerland; 7 Department of Respiratory Medicine, University Hospital Gent, Gent, Belgium; University of California Los Angeles, United States of America

## Abstract

Almost all animal models for chronic rejection (CR) after lung transplantation (LTx) fail to resemble the human situation. It was our attempt to develop a representative model of CR in mice. Orthotopic LTx was performed in allografts receiving daily immunosuppression with steroids and cyclosporine. Controls included isografts and mice only undergoing thoracotomy (SHAM). Allografts were sacrificed 2, 4, 6, 8, 10 or 12 weeks after LTx. Pulmonary function was measured repeatedly in the 12w allografts, isografts and SHAM mice. Histologically, all allografts demonstrated acute rejection (AR) around the blood vessels and airways two weeks after LTx. This decreased to 50–75% up to 10 weeks and was absent after 12 weeks. Obliterative bronchiolitis (OB) lesions were observed in 25–50% of the mice from 4–12 weeks. Isografts and lungs of SHAM mice were normal after 12 weeks. Pulmonary function measurements showed a decline in FEV_0.1_, TLC and compliance in the allografts postoperatively (2 weeks) with a slow recovery over time. After this initial decline, lung function of allografts increased more than in isografts and SHAM mice indicating that pulmonary function measurement is not a good tool to diagnose CR in a mouse. We conclude that a true model for CR, with clear OB lesions in about one third of the animals, but without a decline in lung function, is possible. This model is an important step forward in the development of an ideal model for CR which will open new perspectives in unraveling CR pathogenesis and exploring new treatment options.

## Introduction

The long-term outcome of LTx is hampered by the occurrence of CR. As a consequence, the long-term survival is still inferior compared to other solid organs [1]. CR is morphologically characterized by obliterative bronchiolitis (OB) and clinically by an irreversible decline in forced expiratory volume in one second (FEV_1_), named bronchiolitis obliterans syndrome (BOS). Many animal models have been developed to study OB/BOS going from heterotopic tracheal transplantations [2], [3] to instillation of noxious agents intratracheally [4], [5].

The model most widely used to study OB, the heterotopic tracheal transplantation, has some major shortcomings: the graft is not placed in its physiological environment and is not in contact with air; the trachea is a large airway while OB is per definition small airway pathology and parenchyma and blood vessels cannot be studied since they are absent. Obviously, diagnostic measures such as broncho-alveolar lavage (BAL) and lung function measurements are impossible in this model.

The ideal model to study OB/BOS should mimic the human situation as much as possible, thus: an orthotopic LTx model between major mismatched individuals receiving immunosuppression which leads to histological OB lesions and wherein, ideally, lung function can be measured and BAL can be analyzed. Orthotopic LTx has been described in large animals like dogs [6], swine [7] and primates [8] allowing measurements of physiological parameters but these species are more difficult to house and to handle and are very expensive. Very recently, Jungraithmayr et al. described a new rat model of CR wherein orthotopically transplanted lungs show fibroproliferation of the airways [9]. However, the lack of potential to study mechanistic pathways due to the shortage of specific antibodies, transgenic and knock-out strains of this species may limit their use in the study of BOS pathogenesis. The only species that may hold this potential is the mouse, and, although its size seriously complicates surgery, this species indeed offers a large variety of transgenic strains and available antibodies.

In 2007, Okazaki et al. were the first to describe orthotopic LTx in mice [10]. This technique opened new perspectives in the search for an ideal model for BOS since it mimics the human situation well and BAL and lung function might be used in these animals. The aim of our study was therefore to first develop a murine OB/BOS model after orthotopic LTx in major MHC mismatched mice receiving immunosuppression with OB lesions detectable in the allograft and secondly, to explore the potential of pulmonary function measurements and BAL in this model.

## Methods

### Study Design

**Figure 1:**
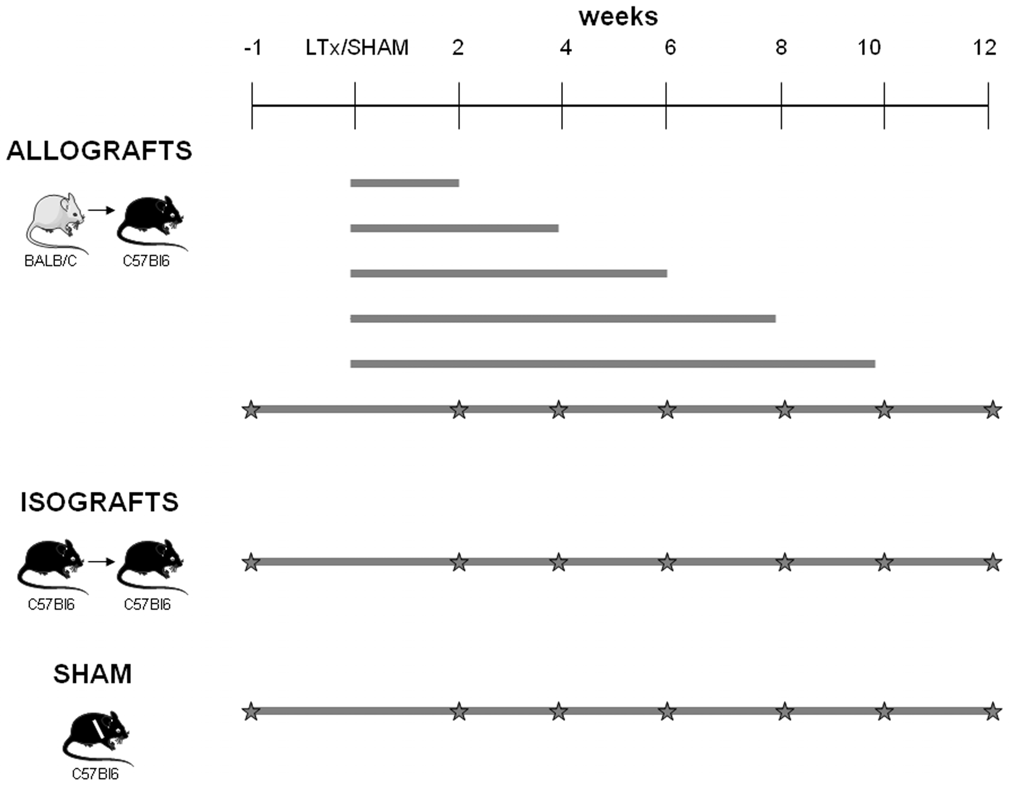
Study design. Allograft: from BALB/C to C57BL6, isografts from C57BL6 to C57BL6. ⋆ represents the time point of lung function measurement, at the end of each time bar animals were sacrificed and BAL and histology were performed.

The study design is illustrated in Figure 1. Since pilot experiments showed clear OB lesions in some allografted mice under immunosuppression, we performed LTx in both allografts and isografts. Allografts were sacrificed after 2, 4, 6, 8 or 10 weeks (n = 5 per group). To explore the potential of lung function measurements (according to the definition of BOS) a separate group of allografts underwent two-weekly, repeated pulmonary function measurements and was sacrificed at 12 weeks after LTx (n = 10). To assess the influence of LTx surgery alone, a group of isografts (n = 9) also underwent two-weekly lung function measurements and were sacrificed afterwards. To determine the influence of the thoracotomy, without LTx, lung function of SHAM mice (n = 9), only undergoing thoracotomy, was also measured repeatedly during 12 weeks.

### Animals

All animals were purchased from Janvier Elevage (France). Male, 25–30 gr C57BL6 mice (H-2K^b^) were used as recipients. Male BALB/C mice (H-2K^d^) served as donors for allografts, C57BL6 mice served as donor for isografts. SHAM mice (C57BL6) only underwent thoracotomy. The protocol was approved by the local Ethics committee (Ethische commissie proefdierencentrum Katholieke Universiteit Leuven, n° 075/2008) and all animals received humane care in compliance with “The Principles of Laboratory Animal Care” formulated by the National Society for Medical Research and the “Guide for the Care and Use of Laboratory Animals” published by the National Institutes of Health (NIH Publication No. 86-23, revised 1996).

### Orthotopic LTx

Orthotopic LTx was adapted from the technique described by Jungraithmayr et al. [11]. Donor mice were anesthetized with a mixture of medetomidine (1 mg/kg) and ketamine (75 mg/kg) intraperitoneally. A tracheostomy was performed and the mouse was connected to a ventilator (UNO microventilator UMV-03, UNO Roestvaststaal, Zevenaar, The Netherlands). Abdomen and thorax were opened and the lungs were flushed with 5 ml Perfadex® (Vitrolife, Göteborg, Sweden) through the arteria pulmonalis. Afterwards, the heart-lung block was excised with inflated lungs and put on ice. The hilum of the left lung was dissected and artery, vein and bronchus were cuffed. Afterwards, the recipient was anesthetized with isoflurane (Isoba®, Schering-Plough, Uxbridge, UK) in an induction box. The animal was intubated and connected to the ventilator with a mixture of 50% O_2_, 50% air and 2.5% isoflurane. After thoracotomy, artery, vein and bronchus were separated from each other and 10-0 ligatures were placed around the structures. Microvascular clamps were put on artery and vein. First the vein was anastomosed, then the artery and subsequently the bronchus. The clamps were released (first vein, then artery) and the lung inflated. Hereafter, the chest was closed and the animal could wake up. Pain medication, with buprenorphine (0.1 mg/kg every 8 hours during 3 days, Temgesic®, Schering Plough, UK) was given as soon as animals regained full consciousness.

### Immunosuppressive treatment

**Figure 2:**
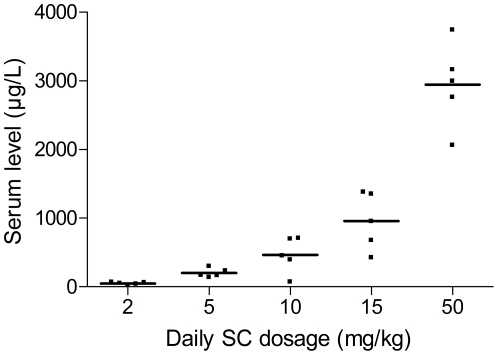
Dose-response curve of Cyclosporine in healthy animals after daily subcutaneous injection during two weeks.

Several pilot experiments were performed to find a balanced immunosuppressive scheme to suppress AR but let OB lesions develop. Different dosages of cyclosporine (5–50 mg/kg/d), based on human clinic and a mouse model of chronic heart rejection [12], were tested until blood levels resembling human blood levels of 200–300 µg/l were obtained. The dose-response curve of different dosages of cyclosporine in healthy animals is illustrated in Figure 2. Since cyclosporine alone was not sufficient to suppress severe AR, steroids were added to the immunosuppressive scheme. Tapering the dosage of steroids and treating the donor with steroids, like in patients, did not make a difference in the severity of AR lesions 2 weeks after LTx. All these experiments led to our current immunosuppressive scheme of 10 mg/kg/d cyclosporine (Sandimmun®, Novartis, Vilvoorde, Belgium)+1.6 mg/kg/d methylprednisolone (Solu Medrol®, Pfizer, Brussels, Belgium) subcutaneously until sacrifice.

### Repeated pulmonary function measurements

FEV_0.1_ in mice was measured repeatedly with the Buxco©-Forced Pulmonary Maneuvers® (Buxco research systems©, Wilmington, North Carolina USA). Intubation and pulmonary function measurement were performed as described previously [13]. Three maneuvers were performed. First the Boyle's law FRC maneuver was performed wherein the animal breathes against a closed valve to measure the Functional Residual Capacity (FRC). Then the quasistatic pressure volume maneuver is performed, wherein the lungs are inflated to 30 cm H_2_O and then slowly exhaled to a negative pressure of −30 cm H_2_O, to measure, amongst others, residual volume (RV) and total lung capacity (TLC). And then the fast flow volume maneuver is performed, wherein the lungs are first inflated to 30 cm H_2_O and then quickly exhaled, which allows the measurement of FEV_0.1_. Pulmonary function was performed one week before LTx/thoracotomy and every two weeks thereafter until 12 weeks after LTx.

### BAL and Histology

Blood was drawn from the vena cava for the evaluation of cyclosporine blood levels (sequential enzyme immunoassay, Dimension® RXL, Siemens Medical solutions, Diamond diagnostics, USA) at time of sacrifice. Immediately afterwards, BAL was performed in left and right lung separately with 4 aliquots of 0.5 ml sterile saline per lung. Total cell counting was performed using the Countess™ Automated Cell Counter (Invitrogen, Merelbeke, Belgium) and cytospins (Shandon) with May-Grünwald-Giemsa staining were made for differential cell counting.

After BAL, the heart-lung block was excised and fixed in 4% paraformaldehyde at a constant hydrostatic pressure of 20–25 cm fluid column for at least 20 minutes and embedded in paraffin. Sections were stained with haematoxylin-eosin to evaluate lung damage and inflammation by a pathologist (E.V.). Sirius Red staining was performed to visualize collagen and fibrosis. Immunohistochemistry for CD3 was performed on a representative sample of each type of lesion. The primary antibody for CD3 (DAKO, Heverlee, Belgium) was applied at a dilution of 1/20. The signal was visualized using 3,3′-diaminobenzidine tetrahydrochloride, producing a brown stain at the site of the reaction.

### Statistical analyses

Statistics were performed with Graphpad Prism Software version 4.1 (San Diego, CA, USA) and SAS 9.1 (SAS Institute, NC, USA). General linear mixed models, one way ANOVA with post hoc Tukey tests, Kruskal Wallis and t-tests were used where appropriate; p<0.05 was considered significant.

## Results

### Survival and general health

**Figure 3:**
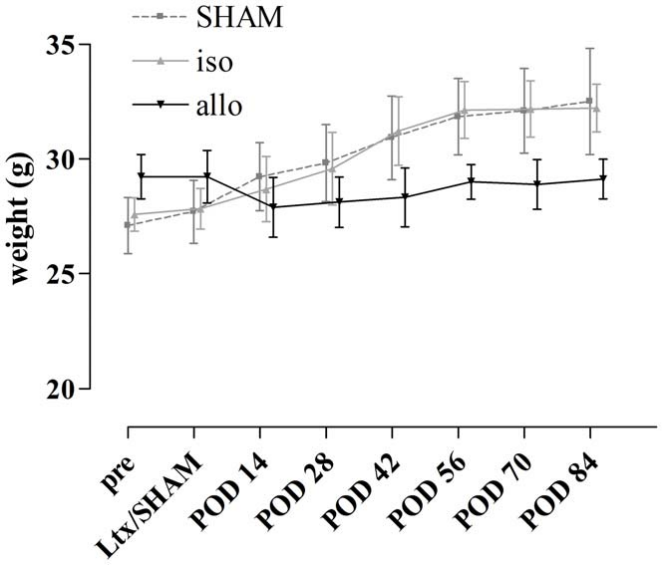
Weight evolution of isografts, allografts and SHAM mice. POD: post-operative day; LTx: lung transplantation; SHAM: only thoracotomy.

Success rate of LTx, with mice succesfully waking up, now reaches up to 90%. No difference in success was noted between isograft and allograft LTx. Weight evolution in the 3 groups is illustrated in Figure 3. Roughly, the weight of SHAM and isografts increased constantly during the post LTx period. Allografts, although somewhat heavier at the beginning of the experiment, lost 1.8 g (0.25–2.05) of bodyweight after the LTx and only regained their initial weight slowly. Body weight increased more in isografts and SHAM mice than in allografts (p<0.0001).

Of the successful operated mice (surviving more than 24 hours), all mice not undergoing pulmonary function measurement survived until sacrifice after 2, 4, 6, 8 or 10 weeks. All SHAM mice survived the consecutive pulmonary function measurements. In the isograft group two mice died: one mouse died after a very difficult recovery from the first pulmonary function measurement after LTx (POD 14), the other mouse died 5 days after the second lung function measurement after LTx (POD 33). In the allograft group 3 mice died during the 12w follow-up: one after a difficult recovery from the second pulmonary function measurement after LTx (POD 29), one each 4 and 5 days after the third measurement after LTx (POD 46 and 47). Survival was comparable between the three groups (p = 0.22).

### Pulmonary function measurements

FEV_0.1_ showed an important decline in the allografts from 0.98 (0.89–1.12) ml before LTx to 0.79 (0.63–0.84) ml two weeks after LTx. In the isografts and SHAM mice the initial decline was very small from 0.78 (0.75–0.88) ml to 0.75 (0.69–0.91) ml in the isografts and from 0.97 (0.85–1.05) ml to 0.96 (0.81–1.07) ml in the SHAM mice. This decline was different between the 3 groups (p = 0.0002) and bigger in allografts than in SHAM mice (p<0.001) and isografts (p<0.001). From then on, FEV_0.1_ of allografts increased and fully recovered after 12 weeks. FEV_0.1_ from both isografts and SHAM mice restored to pre-LTx values within 4 weeks after LTx. After this initial decline we could see a significant difference in FEV_0.1_ evolution between the groups with a bigger FEV_0.1_ increase after LTx in allografts than in the other groups (p = 0.0002).

TLC showed also the biggest decline in the allografts from 1.06 (1.00–1.28) ml before LTx to 0.82 (0.74–0.90) ml two weeks after LTx. Decline in TLC was different in the 3 groups (p = 0.0008). After LTx, TLC followed about the same trends as FEV_0.1_ with again the biggest increase in the allografts (p = 0.0313).

**Figure 4:**
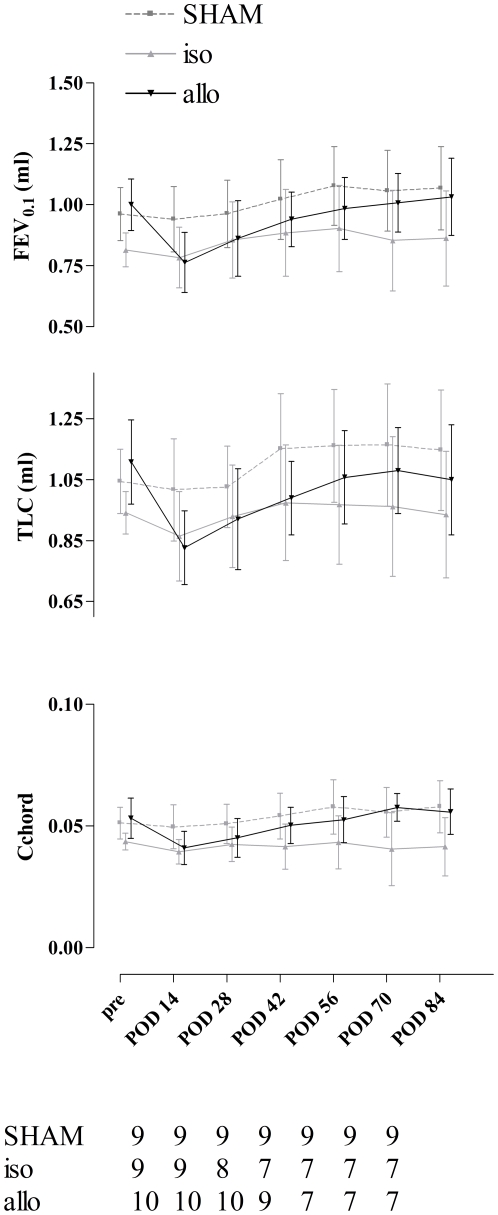
Lung function evolution of the different groups. Decline in FEV_0.1_ and TLC from before transplantation to 2 weeks after transplantation was significantly different between the groups (FEV_0.1_: p = 0.0002; TLC: p = 0.0008) and afterwards the increase in both these parameters was biggest in allografts (FEV_0.1_: p = 0.0002; TLC: 0.0313). FEV_0.1_: forced expiratory volume in 0.1 second; TLC: total lung capacity; Cchord: compliance; POD: post-operative day.

The same was true for compliance (Cchord): biggest decline in the allografts (p = 0.001) and after LTx the biggest increase was seen in the allografts (p = 0.0008). FEV_0.1_, TLC and Cchord evolution of the 3 groups are shown in Figure 4.

### Broncho-alveolar lavage

BAL was performed at time of sacrifice (2–12 weeks after LTx). Total cell counts of the right native lungs were: 432,500 (257,500–597,500) cells/ml in the allografts, 70,000 (55,000–185,000) cells/ml in the isografts and 162,500 (525,00–270,000) cells/ml in the SHAM mice. These differences were statistically significant (p = 0.0008) and post hoc tests showed a significant difference in allografts vs isografts (p<0.01) and allografts vs SHAM (p<0.05). Neutrophil percentages of the right lungs were not statistically different between the 3 groups: 0 (0–4)% for the allografts, 0 (0–6)% for the isografts and 0 (0–0.5) % in the SHAM mice. Macrophage percentages were also not different between the groups (p = NS).

Total cell counts of the left lungs were: 50,000 (20,000–85,000) cells/ml in the isografts and 85,000 (47,250–122,500) cells/ml in the SHAM mice (p = NS). Unfortunately, BAL was technically not possible in most of the allografted left lungs.

### Histology

**Figure 5:**
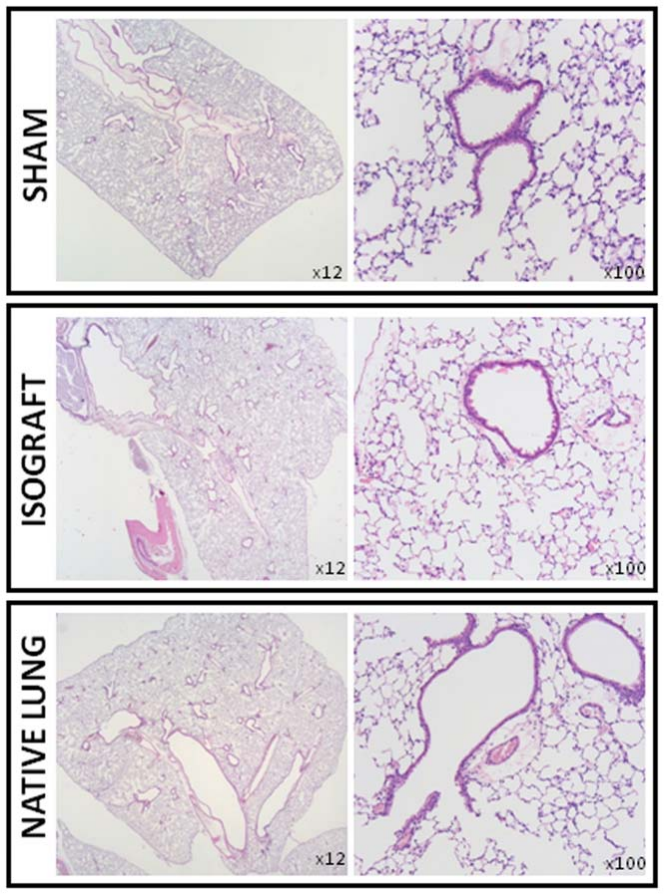
Histology of SHAM mice (top), isografts (middle) and native right lungs from allografts (bottom) 12 weeks after thoracotomy/lung transplantation respectively. HE staining showed normal histology.

As expected, no histological changes were found in SHAM mice (Figure 5). Isografted lungs also looked normal after 12 weeks showing no inflammation, fibrosis etc. (Figure 5). Native right lungs of allografts (and isografts) looked also completely normal (Figure 5).

**Figure 6:**
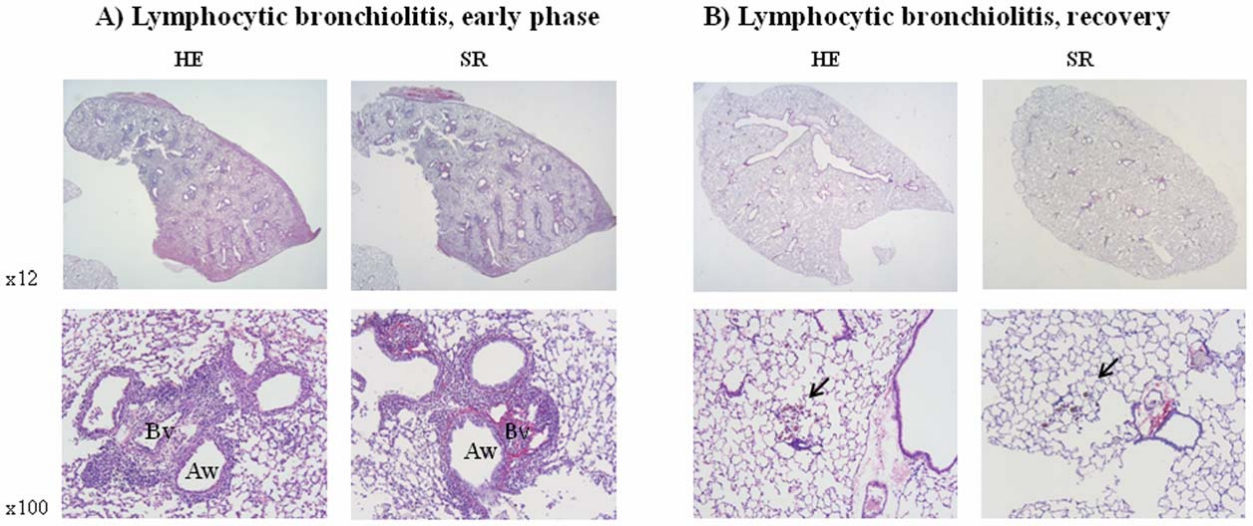
Histology of allografts showing lymphocytic bronchiolitis lesions. A) early after transplantation (2w) and B) late after transplantation (12w). Left side: HE staining, right side: Sirius Red (SR) staining. 2w after transplantation allografts showed enlargement of broncho-vascular axes with infiltration of lymphocytes around airways (Aw) and blood vessels (Bv). Sirius Red staining shows collagen both perivascular as peribronchial. After 12w, lungs completely recovered with some leftover damage seen in pigmented macrophages (arrow).

In the allografts, however, clear histological changes were found and overall, Sirius Red staining showed more collagen in the allografts compared to the native right lungs. After two weeks, inflammation of the broncho-vascular axes, within the walls of vessels and airways, was seen in all allografts. The respective lumina were patent and the inflammatory cells consisted mainly of lymphocytes. The epithelium was preserved and the alveolar parenchyma showed preserved architecture and looked functional (Figure 6). Some oedema, at times some hemorrhage was also seen however. All together, these lesions histologically resemble A and B grade lesions of AR as described by Stewart et al. [14].

**Figure 7:**
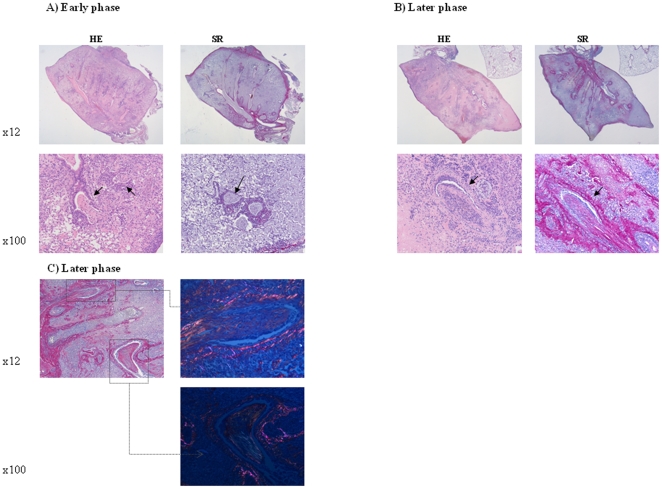
Histology of allografts showing obliterative bronchiolitis. A) early after transplantation (4w) and B+C) late after transplantation (10w). HE staining and Sirius Red (SR) staining are represented in A and B. In the right panel of C polarized SR images are shown. OB lesions are indicated with arrow. Early after transplantation, fibrotic plugs are poor in cells and show little collagen staining. Later after transplantation, cells are more prominently present in the fibrotic plugs and intensive collagen staining of plugs is seen. The polarized images clearly indicate the difference in younger, more reddish collagen and older, more white collagen.

**Figure 8:**
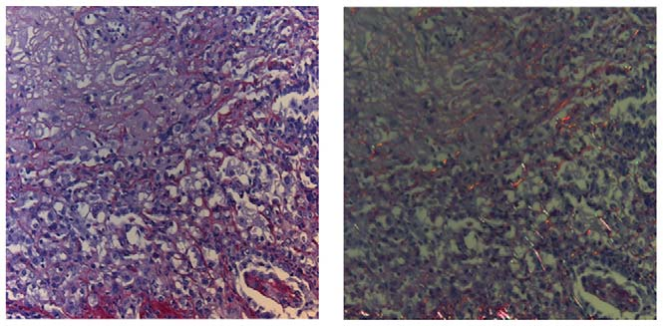
Detail of the parenchyma of allografts showing obliterative bronchiolitis late after transplantation (10w). Left: non-polarized Sirius Red staining; right: polarized Sirius Red. The polarized image clearly shows that fibrosis is absent in the parenchyma. The small amounts of polarized structures indicate native framework.

At later time-points (4–12 weeks) we observed a clear distinction in two types of lesions. One fourth to half of the samples, depending on the time-point, showed very distinct OB lesions with fibrotic plugs growing into the airways. The epithelium of these airways is mainly normal and only damaged where the fibrotic plug is growing into the lumen. The surrounding parenchyma showed postobstructive changes with aspects of endogenous lipid pneumonia with foamy macrophages and interstitial inflammation (Figure 7). Polarized Siriud red images clearly demonstrate the absence of fibrosis in the parenchyma (Figure 8). OB lesions showed clear evolution over time going from immature lesions mainly existing of fibrine without cells to organizing lesions containing cells and angiogenesis to end-stage, epitheliazed lesions without inflammation and with dense collagen staining (Figure 7). Polarization of the Sirius Red slides clearly shows a distinction in younger, more reddish collagen structures and older, more white collagen (Figure 7C).

The other lungs showed AR lesions as described above and over time, inflammation diminished and remarkebly at 12 weeks after LTx 2/3 of the lungs showed normal histology with mimimal rest damage seen in pigmented macrophages (Figure 6).

**Figure 9:**
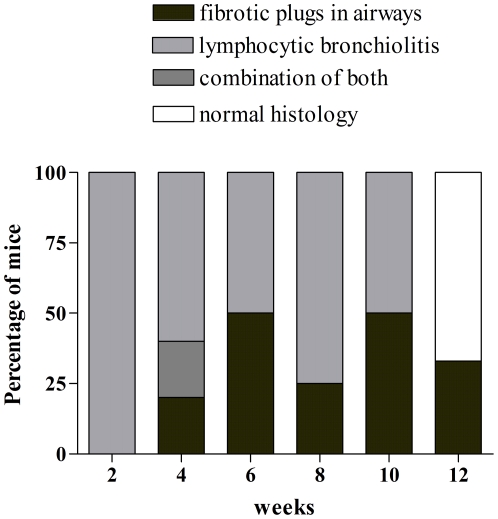
Distribution of the histological lesions of the airways at the different time points after lung transplantation. Perivascular lesions were not taken into consideration.

**Figure 10:**
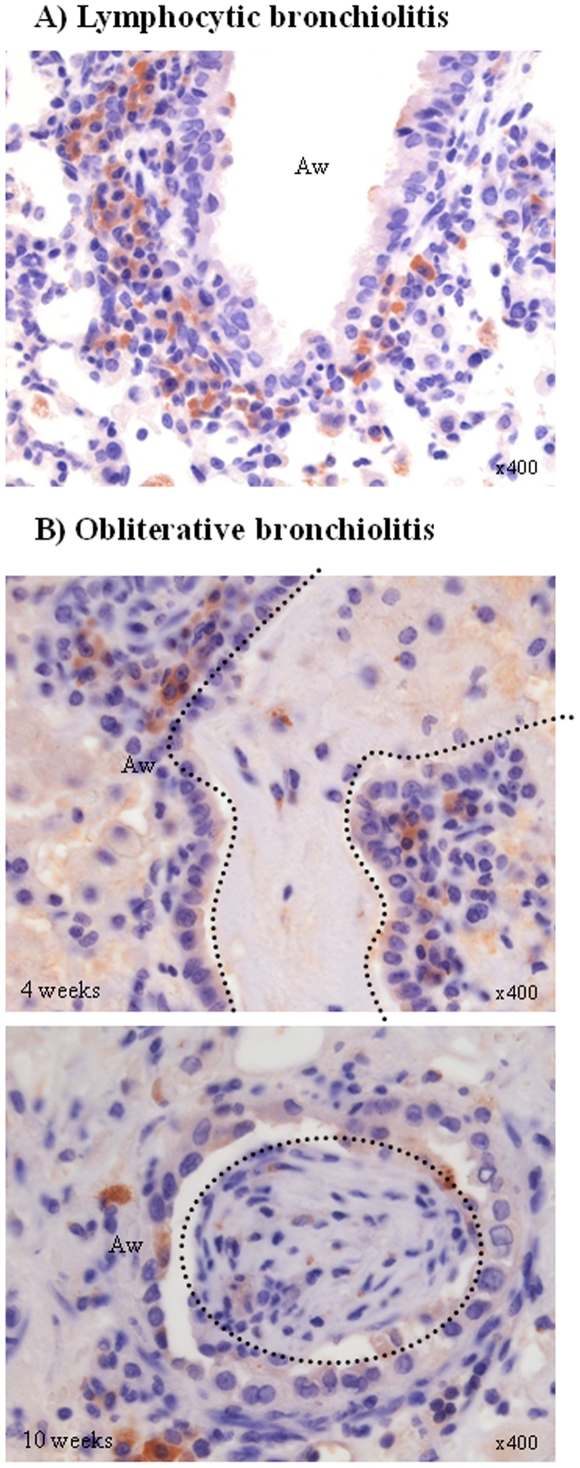
CD3 immunohistochemistry of lymphocytic bronchiolitis and obliterative bronchiolitis lesions. A) lymphocytic bronchiolitis and B) obliterative bronchiolitis lesions both early (4w) and late (10w). Brown cells represent CD3 positive cells. Aw: airway, dotted lines represent approximate contours of the fibrotic plugs.

A combination of both types of lesions, with fibrotic plugs, enlargement of the broncho-vascular axes and functional parenchyma, was seen in one animal. Distribution of the lesions at the different time points is given in Figure 9. CD3 immunohistochemistry confirmed the presence of lymphocytes in the lymphocytic bronchiolitis type of lesions (Figure 10). In the OB lesions an occasional lymphocyte was seen around the airways. Of the 32 animals in these series, 3 mice showed necrosis of the allograft, probably due to an early event. These mice were not taken into account in the distribution of rejection lesions.

### Cyclosporine serum levels

Serum level of cyclosporine was assessed in 22 mice distributed over the different time points. Median serum level of cyclosporine was 392 (248–624) µg/l, comparable to human serum levels. Cyclosporine levels did not differ between time-points after LTx (p = 0.34). However, retrospectively comparing cyclosporine levels in mice showing OB and non-OB lesions showed a significant difference (p = 0.015) with lower cyclosporine levels in the mice showing OB lesions (247 (222–422) µg/l) than in mice showing lymphocytic bronchitis lesions (496 (361–720) µg/l).

## Discussion

The present study was designed to develop a murine model of BOS resembling as much as possible the human situation wherein mice receive a major mismatch graft in which histological OB lesions develop during daily immunosuppression. The potential of repeated pulmonary function measurements and BAL were also examined in this model. Very distinct histological OB lesions were seen in these allografts and repeated lung function measurements were feasible. A true model of BOS with decline in pulmonary function and changes in BAL cellularity was, however, not established. Nevertheless, this model looks very promising in unraveling CR pathogenesis and in exploring new treatment options.

The orthotopic mouse LTx model has so far mainly been used in the study of acute rejection [15] and ischemia-reperfusion injury [16]; [17], both risk factors for BOS. Since BOS is the main cause of late mortality/morbidity after LTx, the development of a new BOS model starting from orthotopic LTx is a logical step. Very recently, Fan et al. were the first to describe OB lesions in allografted mice [18]. The OB lesions described are very similar to those in our model and although proportions of mice showing OB lesions are slightly different, about the same trends are seen with about one fourth to half of the mice showing OB. Histology images of the whole lung, however, are lacking in their paper although these allow the asssessment of lesion distribution, not possible in humans. Also lymphocytic bronchiolitis lesions have not been described in detail while these lesions gained interest in long-term survival after LTx [19]. Some other major differences with our, independently developed, model are present. While they used a minor mismatch combination of mice without immunosuppression, our aim was to really mimic the human situation wherein major mismatch combinations of donor/recipient are predominant and all allografted animals receive immunosuppression. Since mimicking the human situation was our primary goal it was our attempt to develop a true BOS model with BAL and lung function measurements and, for now, not to look at pathological mechanisms.

Several hurdles needed to be taken to develop this model. The surgical technique had to be adopted and a good immunosuppressive scheme needed to be developed since we know from the literature that, if no immunosuppression is given to major mismatched mice, the allograft undergoes severe AR and necrosis after 4 weeks [20].

This study has some shortcomings. 3/32 mice showed necrosis of the graft probably due to malfunctioning of the graft and could not be evaluated in the scope of rejection. Although patients with BOS have an irreversible decline in FEV_1_, this decline could not be reproduced in mice and pulmonary function measurement is not the good tool to diagnose OB lesions since lung volumes restore to normal values over time in allografted mice. The same phenomenon can be seen in mice undergoing a left pneumonectomy. Voswinckel et al. indeed showed that 21 days after left pneumonectomy, mid-expiratory airflow (a parameter for lung volume) reached control values [21]. This demonstrates the enormous growth capacity of the right lung in adult mice. The decline in lung function early after LTx in the allografts, however, demonstrates that lung function measurements may demonstrate signs of AR as the decline in different parameters was much more pronounced in allografts compared to isografts and SHAM mice. The differences in decline early after LTx and in recovery afterwards can partly be explained by a difference in weight between allografts and isografts (and SHAM) at the beginning of the experiment. Mice were transplanted when weighing 25–30 g but an exact weight match between the groups was never aimed for. And since pulmonary function of the mice is measured exactly 1 week before they are being transplanted and only 2 mice a day can be transplanted, day-variability can also partly explain these baseline differences.

BAL was unfortunately not possible in the transplanted allografts, although histology showed open anastomoses (data not shown) and fixating the lung with paraformaldehyde was possible indicating that the anastomoses were indeed open. So far, no conclusion can be drawn about which cells occur in the airways and future experiments should be performed to optimize BAL sampling in these allograft lungs.

The OB lesions we demonstrated, though, were very clear and confirm the feasability of achieving these lesions in transplanted mice. OB lesions occurred in 25–50% of the mice from 4 weeks on, depending on the time-point after LTx. The strength of our study is that we can see a distinct evolution in the OB lesions over time. Lesions evolve from pure fibrinous to active, cell-containing lesions to inactive, end-stage, pure collagenous lesions which stain intensively with Sirius Red. By polarizing the Siriud red stained slides, a distinction in young and older collageneous structures can be seen within these fibrotic plugs demonstrating their evolution. Evolution of these lesions over such a long period of time (12 weeks) has never been described before and this evolution throws new light over the pathogenesis of CR in patients since obtaining consecutive biopsy samples in patients is nearly impossible. A remarkable difference with human OB pathology is that the epithelium in our mouse model is more preserved. In fact, the epithelium is only damaged where the fibrotic plug is growing into the airway lumen while in human OB, epithelial damage has been suggested to be the trigger of the cascade leading to OB [22].

A clear distinction can be made in mice OB lungs (25–50%) and non-OB-lungs (50–75%) and this from 4 weeks after LTx. Looking at the time-distribution of the lesions we saw that at 2 weeks after LTx, all lungs showed the same lesions so the question remains why some mice develop OB and other mice do not develop OB and even regain normal histology after 12 weeks. In patients, different risk factors for BOS have been identified like AR, reflux, Pseudomonas colonization and HLA mismatching [23]. Extrapolating this to mice we can assume that the degree of HLA mismatching is not the determining factor for developing one type of lesion or another since the mismatch is identical in all our animals. The mice are housed in a conventional animal facility meaning that infections and colonizations can not completely be ruled out. However, histology showed no infection of the lungs except in one animal and health status of the mice was negative for all viruses/bacteria/parasites according FELASA (Federation of European Laboratory Animal Sciences Associations) recommendations (www.felasa.eu/recommendations). Two weeks after LTx, all lungs showed AR lesions but later on, only 25–50% of the mice developed CR lesions meaning that the development of AR can not be the only explanation for the development of CR. Looking at cyclosporine serum levels though, a difference was seen with lower cyclosporine levels in mice having OB lesions. Although animals are genetically identical, the animals remain different individuals and pharmacokinetics of cyclosporine seem to be different. This difference might hold the key why not all animals develop OB.

In conclusion, we can say that a murine model for CR after LTx has been developed in one third of our animals with multiple immunosuppressants, but not for true BOS with declining lung function and BAL cellular changes. This is a very important step forward in the development of a good animal model for CR which will certainly help in unraveling the pathogenesis and exploring new treatment options of this complication which significantly hampers long-term survival of LTx patients.
